# Intravenous administration of anakinra in children with macrophage activation syndrome

**DOI:** 10.1186/s12969-021-00585-3

**Published:** 2021-06-29

**Authors:** Omkar Phadke, Kelly Rouster-Stevens, Helen Giannopoulos, Shanmuganathan Chandrakasan, Sampath Prahalad

**Affiliations:** 1 Department of Pediatrics, Emory University School of Medicine, Atlanta, GA USA; 2 Children’s Healthcare of Atlanta, Atlanta, GA USA

## Abstract

**Background:**

Subcutaneous anakinra is an interleukin-1 inhibitor used to treat juvenile idiopathic arthritis. Recent reports suggest anakinra can be a valuable addition to the treatment of COVID-19 associated cytokine storm syndrome and the related multisystem inflammatory syndrome (MIS-C) in children. Herein, we describe our experience with intravenously administered anakinra.

**Findings:**

19 Patients (9 male) received intravenous (IV) anakinra for treatment of macrophage activation syndrome (MAS) secondary to systemic lupus erythematosus (SLE), systemic JIA (SJIA) or secondary hemophagocytic lymphohistiocytosis (sHLH). In most cases the general trend of the fibrinogen, ferritin, AST, and platelet count (Ravelli criteria) improved after initiation of IV anakinra. There were no reports of anaphylaxis or reactions associated with administration of IV anakinra.

**Conclusion:**

Intravenous administration of anakinra is an important therapeutic option for critically ill patients with MAS/HLH. It is also beneficial for those with thrombocytopenia, subcutaneous edema, neurological dysfunction, or very young, hospitalized patients who need multiple painful subcutaneous injections.

## Background

Anakinra is a 17 KD recombinant, non-glycosylated Interleukin-1 (IL-1) receptor antagonist. Subcutaneous (SC) anakinra is used in the treatment of systemic JIA (SJIA) [1, 2]. Anakinra has also been described to be effective in the treatment of macrophage activation syndrome (MAS) secondary to sJIA as well as other rheumatic diseases like systemic lupus erythematosus (SLE) and Kawasaki Disease (KD) [2–5]. Recent reports show anakinra can be effective in secondary hemophagocytic lymphohistiocytosis (sHLH) due to non-rheumatic diseases as well [6]. In some situations, such as thrombocytopenia, subcutaneous edema or in children in intensive care setting, it may be necessary to use intravenous (IV) administration of anakinra instead of SC anakinra. There have only been a few studies that evaluated the pharmacokinetics of IV anakinra in the past [7]. In the present era of COVID-19, high-dose anakinra has been shown to improve outcomes associated with hyper-inflammation observed both with SARS-CoV-2 infections and the newly described multisystem inflammatory syndrome in children (MIS-C) [8–10]. We sought to describe our experience with IV anakinra in children with MAS at our institution prior to COVID-19 in order to guide clinicians wishing to consider this therapy for indications such as hyperinflammation seen with COVID-19 and MIS-C.

## Methods

In collaboration with our hospital pharmacists, a protocol was designed for the use of IV anakinra in our center. The protocol outlines potential use of IV anakinra in patients with an underlying rheumatic condition (such as SJIA, SLE, KD) with features of MAS or secondary HLH. These patients may require high doses of anakinra, require multiple subcutaneous injections, may have subcutaneous edema, thrombocytopenia, or coagulopathy. For patients naïve to anakinra, the dose was started at 2 mg/kg and titrated up to a maximum of 100 mg IV every 12 h according to the patient’s clinical status. For patients already on maintenance anakinra and admitted to hospital, anakinra was titrated up to a maximum of 100 mg IV every 6 h according to the patient’s clinical status. The SC formulation was mixed in normal saline with 1 ml of normal saline per 1 mg of anakinra, administered IV over 30 min.

An IRB-approved retrospective chart review of various clinical and demographic variables via electronic medical record were identified for patients that had received IV anakinra at our institution between January 2017 and December 2019.

The duration of therapy, doses and outcome of the patients were recorded. MAS laboratory values as described by the Ravelli criteria [11] were identified prior to and 24 to 48 h after conclusion of administration of IV anakinra.

## Findings

In all, 19 patients (9 male) received IV anakinra (Table 1). All patients met the 2016 Ravelli criteria for MAS [11], except patient #1 and #5, in whom a clinical decision was made to start anakinra due to rising ferritin and transaminases. Eleven patients were in a critical care setting during administration. Median age of our cohort was 13 years. Indication was MAS secondary to SJIA (*n* = 10), SLE (*n* = 3), sHLH (*n* = 5) and other (*n* = 1). All 5 patients with sHLH met 2004 HLH criteria [12] (Table 2). Maximum duration of therapy was 85 days. Median duration of therapy was 10 days. The initial dose of IV anakinra ranged from 1.7 to 10 mg/kg/day and the maximum dose of IV anakinra ranged from 4.2–15.4 mg/kg/day. One patient (#4) was already on 100 mg SQ Q12 of anakinra at home, and this was increased to 100 mg Q6 IV (20 mg/kg/day) to successfully treat an acute episode of MAS. The maximum frequency of administration was every 6 h. In most cases the general trend of the fibrinogen, ferritin, AST, and platelet count improved after initiation of IV anakinra. There were no reports of anaphylaxis or reactions associated with administration of IV anakinra.

**Table 1:** Clinical and laboratory characteristics of patients that received intravenous anakinra

Patient	Age	Sex	Diagnosis	Anakinra dose (mg/kg/d) Initial Max		Duration (Days)	Triglycerides (mg/dl) baseline	Ferritin (ng/dl) Pre-Post		AST (U/L) Pre-Post		Fibrinogen (mg/dl) Pre-Post		Platelets (1000/UL) Pre post	
1	1	M	SJIA	4	7.2	3	109	***4667***	2132	***53***	52	504	253	568	505
2	3	F	SJIA	7.4	7.4	11	***160***	***130,000***	3486	***164***	45	***168***	202	***46***	70
3	4	M	SJIA	7.8	15.4	5	102	***8663***	1097	***766***	160	***81***	170	***84***	173
4	6	M	SJIA	20	20	12	Not done	***10,437***	137	***168***	24	***196***	126	211	265
5	8	F	SJIA	8	8	3	83	***5160***	1954	***74***	472	426	392	343	591
6	13	F	SJIA	3.3	7.5	10	***197***	***21,442***	1442	***935***	42	***83***	152	***113***	267
7	13	F	SJIA	11	11	8	***249***	***55,000***	3933	***325***	107	***118***	91	***120***	311
8	16	M	SJIA	9.4	9.4	5	***207***	***2016***	294	16	13	637	509	471	429
9	16	M	SJIA	6	6	2	***176***	***17,033***	3850	***125***	120	***221***	221	***180***	243
10**	20	F	SJIA	1.7	6.8	5	152	***84,000***	10	***1812***	314	***170***	319	***23***	81
11	16	F	Lupus	2.5	8	47	***268***	***12,098***	412	***432***	6	***323***	214	***139***	150
12	16	M	Lupus	6.6	6.6	10	***165***	***5195***	557	***361***	40	362	607	***134***	287
13	20	M	Lupus	10	10	85	***782***	***120,000***	1421	***2521***	23	***190***	158	239	188
14**	13	M	Vasculitis	7.1	14.2	54	***203***	***3186***	12,398	46	128	***321***	779	***22***	42
15	3	F	sHLH	4	20	16	***333***	***110,000***	1240	***66***	28	***335***	155	298	290
16	9	M	sHLH	3	11	13	***209***	***15,750***	7855	41	42	***295***	405	709	174
17**	10	F	sHLH	4	8	2	***2617***	***1216***	15,577	***270***	394	674	489	***19***	99
18**	12	F	sHLH	2.2	4.2	9	***336***	***92,000***	67,000	***265***	26	375	595	***12***	101
19**	19	F	sHLH	8	8	20	***272***	***13,756***	8004	***166***	134	376	205	***60***	56

Ravelli criteria include Ferritin> 684 ng/dl plus any 2/3 of TG > 156 mg/dl, PLT < 181 (1000/UL), AST > 48 U/L, Fibrinogen< 360 mg/dl. Values meeting these criteria shown in *italics* and **bold**. All patients except patient #1 and #5 met Ravelli criteria 2016, who had elevation in ferritin and elevated AST only

Patient with ** (Patient #10, #14, #17, 18# and #19) are deceased

*AST* Aspartate aminotransferase, *sJIA* Systemic Juvenile Idiopathic Arthritis, *sHLH* secondary hemophagocytic lymphohistiocytosis

**Table 2:** Features of patients meeting HLH 2004 criteria

	Patient 15	Patient 16	Patient 17	Patient 18	Patient 19
Familial Genetic Panel	Negative	Negative	Heterozygous mutation: **UNC13D** C753 + 1 G > T	Negative	Heterozygous mutations: ***STXBP2*** T248M ***LYST*** R3412H
Fever > 7 days	Yes	Yes	Yes	Yes	Yes
Splenomegaly	No	No	No	Yes	No
Cytopenia’s (> 2 lineages)^x^	No	No	Yes	Yes	Yes
Hypertriglyceridemia (> 265 mg/dl) or Hypofibrinogenemia (< 150 mg/dL)	Yes	Yes	Yes	Yes	Yes
Hemophagocytes on bone marrow	Yes	Yes	Not done	Yes	No
Low NK cell activity	No	Yes	Yes	Yes	No
Ferritin > 500 micrograms/ L	Yes	Yes	Yes	Yes	Yes
Soluble CD25 > 2400 U/mL	Yes	Yes	No	No	Yes

Cytopenia ^x:^ Hemoglobin < 9 g/dL, Platelets < 100 × 10^9^ /L or Neutrophils < 1 × 10^9 /L^

Patients meeting HLH 2004 criteria. To fulfil HLH 2004 criteria patients had to meet at least 5 of 8 criteria. All five patients met criteria

Patient #17 and #19 had heterozygous mutations in UNC13D, STXBP2 and LYST genes. These genes have been shown to harbor pathogenic variants related to Hemophagocytic Lymphohistiocytosis

Increased transaminases were noticed in patient #5 who received a maximum dose of 8 mg/kg/day (224 mg); discontinuation of anakinra resulted in normalization of AST and ALT. Five (26.3%) of the patients died from their underlying disease or complications. Other medications received by patients who died are depicted in Table 3**.** Patient #10 had SJIA and MAS; MAS laboratory parameters improved after IV anakinra administration. However, she developed Methicillin sensitive *Staphylococcus aureus* bacteremia (MSSA) leading to multi organ failure and cardiorespiratory arrest. Patient #14 had recurrent refractory ischemic strokes secondary to vasculitis of unknown etiology and multi-organ failure with MAS. Patient #17 with primary immune dysregulation (mutation in MUNC 13) died of overwhelming cytomegalovirus viremia (CMV) and MSSA bacteremia. Patient #18 with refractory HLH and CNS involvement also had overwhelming sepsis. Patient #19 with HLH status post BMT and recurrent CMV viremia died from multi organ failure, however anakinra had been used a year prior to her death. Three of these patients (#10, #18, #19) had improvement in the Ravelli MAS laboratory parameters in response to IV anakinra despite their fatal outcome.

**Table 3:** Other medications received by selected patients

Patient number	Diagnosis	Other immunosuppressive medications used during admission	Outcome
10	SJIA	Methylprednisolone, Etoposide, Dexamethasone, Jakafi	Deceased
14	Unclassified Vasculitis	Cyclophosphamide, Rituximab, Eculizumab	Deceased
17	sHLH	Ruxolitinib, Methylprednisolone	Deceased
18	sHLH	Dexamethasone, Cyclosporin, Etoposide	Deceased
19	sHLH	Mycophenolate, Steroids, Eculizumab	Deceased
15	sHLH	Dexamethasone, Etoposide	Recovered
16	sHLH	Dexamethasone, Etoposide	Recovered

Other medications received in selected patients (deceased and/or those with sHLH) during the admission at which IV anakinra was used

## Discussion

Our experience with IV anakinra administration prior to the recent COVID-19 pandemic indicates that intravenously administered anakinra was overall safe and well tolerated with minimal adverse effects apart from one case of elevated transaminases, which is a known side effect of SC anakinra and described by Canna et al. [13]. There were no reported instances of anaphylaxis. It was effective for the treatment of MAS with improvement of laboratory parameters in most instances. Thus, IV anakinra may be an important therapeutic option for critically ill patients, although there is limited literary evidence regarding the pharmacokinetics, absorption, and efficacy of IV anakinra.

Prior studies of IV anakinra in sepsis have not shown an increase risk of adverse effects. In 1994, Fisher et al. [14] reported no statistically significant increase in survival time for Interleukin-1 antagonist treatment compared with placebo among all patients who received the study medication or among patients with sepsis. In a multicenter trial in 1997, Opal et al. [15] failed to demonstrate a statistically significant reduction in mortality when continuous IL-1 receptor antagonist infusions were compared with standard therapy in sepsis. In both these instances no excess adverse effects or microbial superinfections were attributed to the IL-1 inhibitor [14, 15]. In a large cohort of 763 patients, Shakoory et all in 2016 showed significant clinical improvement with treatment of IV anakinra vs placebo for sepsis patients with features of MAS [16].

Mehta et al. recently described high dose IV anakinra for MAS/HLH in cytokine storm syndromes [10]. They used IV anakinra in 39% of their patient population with cytokine storm and no adverse effects were seen. Cavalli et al. used IV anakinra at 10 mg/kg/day and showed improvement in COVID-19 associated hyperinflammation in 72% of their cohort [8]. Montegudo et al. used continuous Anakinra (2400 mg/day) in treatment of MAS/ sHLH with clinical improvement in 4/5 patients [17]. In a recent paper (December 2020) Kavirayani et al. successfully used IV anakinra at extremely high doses (48 mg/kg/day) for the treatment of non- familial CNS HLH even in the setting of intercurrent infections [18]. Thus, IV anakinra could be an option for treating COVID-19 associated hyperinflammatory state and/or cytokine storm in selected patients and appears to be well tolerated at high doses and in the setting of sepsis.

Limitations of our study include that it was retrospective in nature and only a relatively modest number of patients were included. However, we believe these cases are illustrative regarding the use of IV anakinra. Fatal outcome was observed in five patients in our series (26.3%) similar to findings noted by Eloseily et al. [19]. These patients either had severe, refractory disease or were immunosuppressed prior to IV anakinra exposure due to other medications/ post bone marrow transplant. Despite this, the laboratory indicators of MAS improved in three of the patients who succumbed to their illness.

## Conclusion

In summary, intravenous administration of anakinra is an important therapeutic option for critically ill patients with MAS/HLH. It is also beneficial for those with thrombocytopenia, subcutaneous edema, neurological dysfunction, or very young, hospitalized patients who need multiple painful injections**.**

## Data Availability

All data generated or analyzed during this study are included in this published article.

## Abbreviations

MISC: Multisystem inflammatory syndrome (MISC)
SJIA: Systemic JIA
MAS: Macrophage Activation Syndrome
sHLH: Secondary Hemophagocytic lymphohistiocytosis
SC: Subcutaneous
KD: Kawasaki disease
CMV: Cytomegalovirus
MSSA: Methicillin sensitive *Staphylococcus aureus* bacteremia
IL-1: Interleukin-1
PLT: Platelets
SLE: Systemic lupus erythematosus
